# Genetic polymorphisms as prognostic factors for recurrent kidney stones: A systematic review and meta-analysis

**DOI:** 10.1371/journal.pone.0251235

**Published:** 2021-05-06

**Authors:** Widi Atmoko, Putu Angga Risky Raharja, Ponco Birowo, Agus Rizal Ardy Hariandy Hamid, Akmal Taher, Nur Rasyid

**Affiliations:** Department of Urology, Faculty of Medicine, Universitas Indonesia, Cipto Mangunkusumo Hospital, Jakarta, Indonesia; Shanghai Jiao Tong University, CHINA

## Abstract

Genetic polymorphisms have been suggested as risk factors affecting the occurrence and recurrence of kidney stones, although findings regarding the latter remain inconclusive. We performed this systematic review and meta-analysis to clarify the associations between genetic polymorphisms and recurrent kidney stones. PubMed, SCOPUS, EMBASE, and Cochrane Library databases were searched through May 28^th^, 2020 to identify eligible studies. The Quality in prognostic studies (QUIPS) tool was used to evaluate bias risk. Allelic frequencies and different inheritance models were assessed. All analyses were performed using Review manager 5.4. A total of 14 studies were included for meta-analysis, assessing urokinase (ApaL1) and vitamin D receptor (*VDR*) (ApaI, BsmI, FokI, and TaqI) gene polymorphisms. The ApaLI polymorphism demonstrated protective association in the recessive model [odds ratio (OR) 0.45, *P* < 0.01] albeit higher risk among Caucasians in the heterozygous model (OR 16.03, *P* < 0.01). The VDR-ApaI polymorphism showed protective association in the dominant model (OR 0.60, *P* < 0.01). Among Asians, the VDR-FokI polymorphism recessive model showed significant positive association (OR 1.70, *P* < 0.01) and the VDR-TaqI polymorphism heterozygous model exhibited protective association (OR 0.72, *P* < 0.01). The VDR-BsmI polymorphism was not significantly associated with recurrent kidney stones in any model. Urokinase-ApaLI (recessive model), VDR-ApaI (dominant model), and VDR-TaqI (heterozygous model) polymorphisms were associated with decreased recurrent kidney stone risk whereas urokinase-ApaLI (heterozygous model) and VDR-FokI polymorphisms were associated with increased risk among Caucasians and Asians, respectively. These findings will assist in identifying individuals at risk of kidney stone recurrence.

## Introduction

A global increase in the incidence and prevalence of kidney stones has been documented in almost all populations, with the prevalence rates varying from 1 to 20% [1]. In the United States, the lifetime risk of kidney stones is 8.8% with approximate recurrence rates of 14 and 35% after one and five years, respectively [2, 3]. Moreover, a “stone forming belt” has been recognized in Asia extending from West Asia to Southeast and South Asia with prevalence rates between 5 and 19.1% [4] and lifetime recurrence risks estimated at 60–80% among Asian countries [4]. Notably, the financial burdens of recurrent stone disease are substantial, with. estimates of direct and indirect costs to treat patients with kidney stones exceeding $5 billion USD [5]. Thus, preventing recurrences among at-risk stone formers may constitute a cost-effective approach to manage this disease.

Kidney stones are defined as a complex disease, as they develop from interactions between multiple environmental and genetic factors [6, 7]. In particular, genetic variants in the population may contribute significant risk for manifesting such multifactorial disorders including kidney stone formation and recurrence [8, 9]. It is thus expected that the study of associated genetic polymorphisms will assist in identifying individuals at risk of kidney stone recurrence, defining the pathophysiological mechanisms, and determining novel targets for drug therapy [10]. However, although several genes associated with recurrent kidney stones have been identified, the results have been inconsistent across studies. Additionally, to our knowledge, a meta-analysis evaluating the associations between genetic polymorphisms and recurrent kidney stone has not been reported. Therefore, we performed a systematic review and meta-analysis to comprehensively appraise the existing literature for possible associations between genetic polymorphisms and recurrent kidney stones.

## Material and methods

### Literature search

The Preferred Reporting Items for Systematic Reviews and Meta-Analyses (PRISMA) guidelines were used as guidance to perform this systematic review and meta-analysis [11]. Systematic searches using PubMed, SCOPUS, EMBASE, and Cochrane Library databases through July 19^th^, 2020 were performed to find relevant literature evaluating association between recurrent kidney stones and gene polymorphisms. The following subject terms and keywords were applied: “kidney stone” or “kidney calculi” or “urolithiasis” or “nephrolithiasis” or “urinary calculi” and “genome” or “genetic” or “mutation” or “single nucleotide polymorphism”. Detailed information regarding the keywords and search hits is presented in Table 1. Manual searches were also conducted from references in related studies. The protocol was also registered under PROSPERO database (PROSPERO 2020 CRD42020191348).

**Table 1:** Keywords and search hits in PubMed, EMBASE, SCOPUS, and Cochrane Library.

Search Engine	Search Terms	Number of Articles
Pubmed	(kidney stone[Title/Abstract] OR urolithiasis[MeSH Terms] OR nephrolithiasis[MeSH Terms] OR kidney calculi[MeSH Terms] OR urinary calculi[MeSH Terms]) AND (genome[MeSH Terms] OR genetic[MeSH Terms] OR mutation[MeSH Terms] OR (single nucleotide polymorphism[MeSH Terms]))	517
SCOPUS	TITLE-ABS-KEY(("kidney stone" OR urolithiasis OR nephrolithiasis OR "kidney calculi" OR "urinary calculi") AND (genome OR genetic OR mutation OR "single nucleotide polymorphism")) AND (LIMIT-TO (EXACTKEYWORD, "Human"))	2,403
EMBASE	(’kidney stone’:ti,ab,kw OR urolithiasis:ti,ab,kw OR nephrolithiasis:ti,ab,kw OR ’kidney calculi’:ti,ab,kw OR ’urinary calculi’:ti,ab,kw) AND (genome:ti,ab,kw OR genetic:ti,ab,kw OR mutation:ti,ab,kw OR ’single nucleotide polymorphism’:ti,ab,kw) AND ’human’/de	1,265
Cochrane Library	((kidney stone):ti,ab,kw OR (urolithiasis):ti,ab,kw OR (nephrolithiasis):ti,ab,kw OR (kidney calculi):ti,ab,kw OR (urinary calculi):ti,ab,kw) AND ((genome):ti,ab,kw OR (genetic):ti,ab,kw OR (mutation):ti,ab,kw OR (single nucleotide polymorphism):ti,ab,kw)	39

### Inclusion and exclusion criteria of study selection

The population of interest comprised patients with recurrent kidney stones, defined as a minimum of two symptomatic kidney stone occurrences within at least a six-month interval. Healthy subject groups or patients with a single kidney stone episode were included as comparison groups. The outcome in the eligible studies had to include any genetic polymorphisms associated with recurrent kidney stones. Eligible studies were required to fulfill the following inclusion criteria: (1) cross-sectional, case-control, or cohort studies evaluating the relationship between genetic polymorphisms and recurrent kidney stones; (2) availability of genotype frequency in both case and control groups to allow comparison for calculating odds ratios (OR) and 95% confidence intervals (95% CI); and (3) English language. The following were exclusion criteria in our study: (1) case reports, qualitative studies, in vitro experiments, animal studies, conference or poster abstracts, systematic reviews, and meta-analyses; (2) insufficient demographic data of the population (i.e., kidney stone recurrence was not mentioned); and (3) unavailability of detailed data or full text. Publication dates were not limited. In the case of duplicated studies from the same population, only the study with the largest sample was included.

### Quality assessment and data extraction

Two investigators (W.A. and P.A.R.R) independently performed quality assessment and data extraction. Disagreement was resolved by discussion and the assistance of other investigators if needed. Quality assessment was accomplished by evaluating bias using the Quality in Prognostic Studies (QUIPS) tool [12]. We recognized six domains of bias assessment including: (1) study participation, (2) study attrition, (3) prognostic factor measurement, (4) outcome measurement, (5) study confounding, and (6) statistical analysis and reporting [12, 13]. Each domain was then assigned as having a low, moderate, or high risk of bias [13]. Overall risk of bias was determined based on the ratings of each domain. Information on all eligible studies regarding author, publication year, demographic characteristics, control groups (healthy subjects or first-time stone former), sample size, genotyping method, and genotype distribution was extracted. The Checklist for Critical Appraisal and Data Extraction for Systematic Reviews of Prediction Modelling studies (CHARMS) was used to facilitate the data extraction process [14].

### Statistical analysis

Meta-analysis was performed only for genetic polymorphisms with at least three available studies as a large number of genetic polymorphisms were reported. Associations between genetic polymorphisms and recurrent kidney stones were measured using ORs and 95% CIs. The ORs were calculated for the following five models: (1) allelic frequencies (X-allele vs. x-allele); (2) homozygous (XX vs. xx); (3) heterozygous (Xx vs. XX + xx); (4) dominant (XX + Xx vs. xx; and (5) recessive (XX vs. Xx + xx). A Chi-square-based Q test was performed to check the heterogeneity of the involved studies. Heterogeneity was considered to exist if the *P* value was < 0.10. The fixed-effects model was used to calculate pooled ORs when the studies were homogeneosus (*P* value of Q test > 0.10). In the case of heterogeneity, the random-effects model was adopted. In addition to comparison with healthy subjects, we also performed comparisons with first-time stone formers if at least two studies were available. We also performed sub-analysis on possible sources of heterogeneity, especially ethnicity [15]. Sub-analysis of ethnicity (Asian and Caucasian) was performed if at least two studies were available. Begg’s funnel plot was used to evaluate the publication bias. We also performed meta-analysis with restriction of larger studies (N > 200 cases) for quality control of publication bias [16]. Larger studies are less prone to publication bias compared to smaller studies [16]. The Hardy–Weinberg equilibrium (HWE) in the control group was calculated by using the chi-square test for goodness of fit (chi-square score >3.84 was determined as indicating discrepancy from the equilibrium) [17]. All statistical analyses were performed using Review manager 5.4. software (Revman Cochrane, London, UK).

## Results

### Literature search and study characteristics

**Fig 1:**
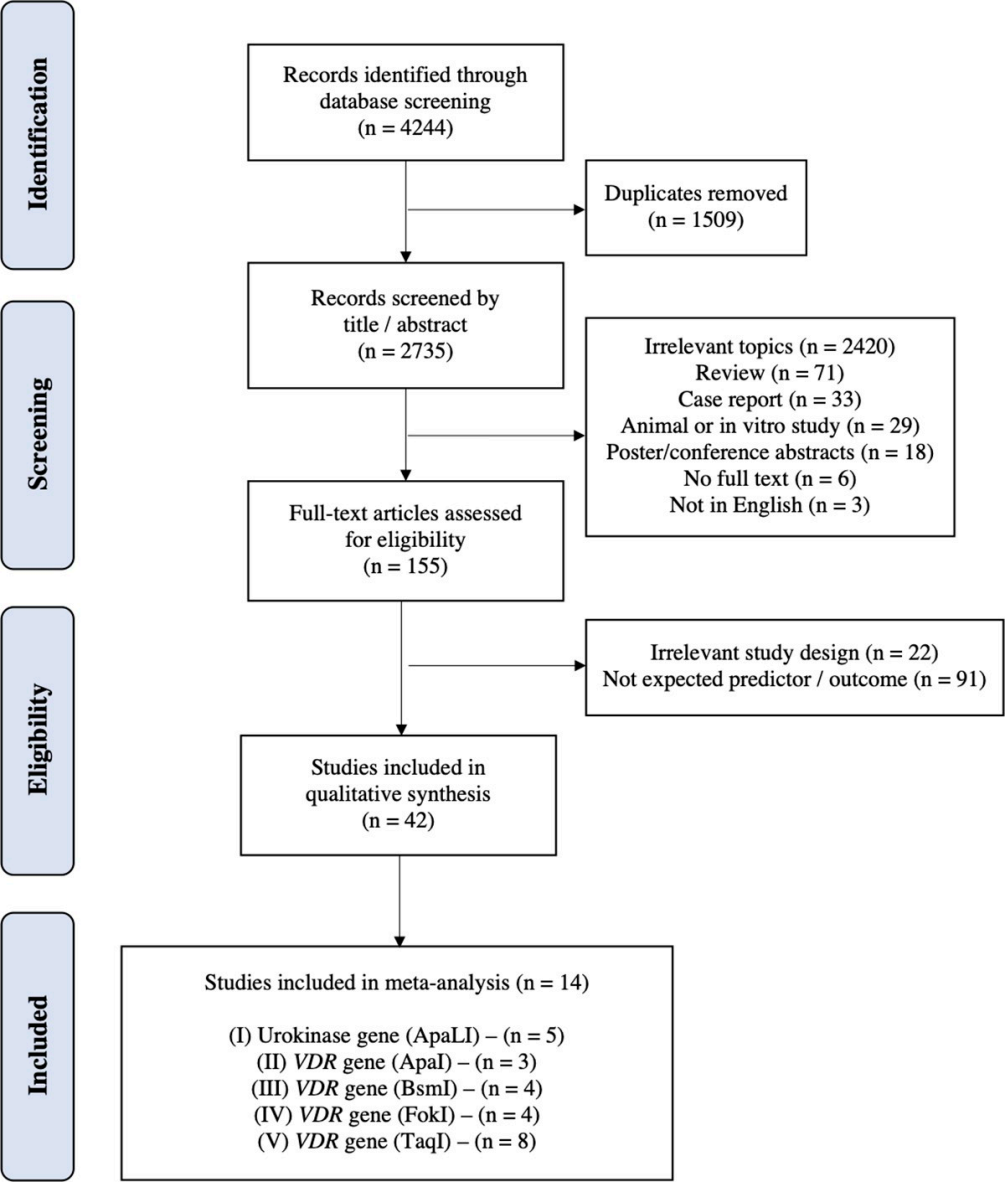
Flow chart of eligible study selection.

The first literature search consisted of 4244 articles of which 2735 remained after removal of duplicates. After screening titles and abstracts, 2420 articles were removed because of irrelevant topics. Furthermore, 71 review articles, 33 case reports, 29 animal or in vitro studies, and 18 poster or conference abstracts were excluded and an additional six articles were excluded because of full text unavailability. Three articles were not written in English. The full text of the remaining 155 articles was then assessed for eligibility. A total of 113 articles were subsequently excluded because of irrelevant design or unavailability of the expected predictor or outcomes. Finally, we identified 42 studies that fulfilled the inclusion criteria for systematic review. Among these, 14 studies were selected for meta-analysis, which evaluated polymorphisms in the urokinase and vitamin D receptor (*VDR*) genes. Specifically, five studies evaluated the urokinase (ApaL1) gene polymorphism [18–22]; for *VDR*, three studies investigated the ApaI polymorphism [7, 23, 24], four each examined the BsmI [23–26] and FokI polymorphisms, [24, 26–28] and eight studies evaluated the TaqI polymorphism [18, 19, 23, 25–27, 29, 30]. The eligibility pathway in our meta-analysis is shown in Fig 1.

In addition, several polymorphisms associated with kidney stone recurrence were only evaluated in a single study, such as in the androgen receptor (*AR*) gene [cytosine, adenine, guanine (CAG) repeat] [31], calcitonin receptor (*CALCR*) gene [AluI, IVS1-6T>C, IVS1insA, IVS5-17(GTTT)3, IVS6A>G, IVS6G>A, IVS10+35-37delT, Ex13T>C, Ex13A>C, and 3′UTR+18C>T] [32, 33], calcium-sensing receptor (*CASR*) gene (A986S, E1011Q, R990G, rs1048213, rs1501899, rs17251221, rs6776158, rs7648041, rs7648044, rs7652589, and rs7627468) [34–37], e-cadherin (*CDH1*) gene (PmlI) [38], cytochrome P450c17α enzyme (*CYP17*) gene (MspAI) [39], epidermal growth factor receptor (*EGFR*) gene (BsrI) [39], estrogen receptor (*ER*) gene [thymine-adenine (TA) dinucleotide repeat] [31], insulin-like growth factor-2 (*IGF2*) gene (ApaI) [39], interleukin-18 (*IL18*) gene (–607C/A, –137G/C, and +105A/C) [40], IL-1 receptor antagonist (*IL1RA*) gene (intron 2 variable number of tandem repeats) [41], *IL1B* gene (promoter, exon 5, and rs16944) [41, 42], *IL6* gene (rs1800795, rs1800796, and rs1800797) [42], inositol 1,4,5-trisphosphate 3-kinase C (*ITPKC*) gene (rs11673492, rs28493229, rs7257602, rs7251246, rs890934, rs10420685, rs2607420, and rs2290692) [43], manganese superoxide dismutase (*Mn-SOD*) gene (BsaWI) [44], melatonin receptor 1A (*MTNR1A*) gene (rs2119882, rs2375801, rs13140012, and rs6553010) [45], osteopontin (*OPN*) gene [9402 (Arg/His) and rs1126616] [46, 47], Ca release-activated calcium channel protein 1 (*ORAI1*) gene (rs12313273) [48], ornithine decarboxylase (*ODC*) gene (+316 G/A) [49], osteocalcin gene (HindIII) [50], renal sodium-citrate (dicarboxylate) cotransporter (hNaDC1; *SLC13A2*) gene (I550V [51], rs11567842 [29]), regulator of G protein signaling 14 (*RGS14*) gene (rs12654812) [34], spermidine/spermine N1-acetyltransferase 1 (*SAT1*) gene (−1415 T/C) [49], transporter associated with antigen-processing (*TAP*) gene (DpnII, AccI, BstUI, MspI, and RsaI) [52], tumor necrosis factor-alpha (*TNFA*) gene (−308 A/G) [53], transient receptor potential cation channel, subfamily V, member 5 (*TRPV5*) gene (rs4236480) [9], *VDR* gene (NT-029419.12: g.10416049C>T) [27], and vascular endothelial growth factor (*VEGF*) gene (BstUI) [39]. Moreover, two studies each were identified that evaluated associations of the alkaline phosphatase, liver/bone/kidney (*ALPL*) gene (rs1256328) [34, 54] and matrix Gla protein gene (rs4236) [55, 56] polymorphisms with recurrent kidney stones. However, these studies were not included in the quantitative synthesis.

Blood samples were used for DNA extraction in all studies chosen for meta-analysis and control groups were primarily matched based on sex and age. However, genotype distributions in the control groups were inconsistent with HWE in several studies. Detailed characteristics of studies that were included in the quantitative synthesis are shown in Table 2. Evaluation using the QUIPS tool revealed that most selected studies showed low-risk of bias, with only two presenting moderate-risk of bias as indicated in Table 3.

**Table 2:** Characteristics of individual studies included in the meta–analysis.

**Study (Urokinase ApaLI)**	**Country**	**Ethnicity**	**Age group**	**Genotyping method**	**Controls**	**Cases (n)**			**Controls (n)** ^**a**^				**Bias Risk (QUIPS)**
						**CC**	**CT**	**TT**	**CC**	**CT**	**TT**	**X**^**2**^ **HWE**	
Aykan et al., 2016 [18]	Turkey	Caucasian	Adult	PCR-RFLP	Healthy subjects & 1st-time stone formers	64	5	9	155	0	12	167 ^b^	Low
Jawad et al., 2020 [19]	Iraq	Asian	Adult	PCR-RFLP	Healthy subjects & 1st-time stone formers	20	16	84	45	40	42	17.4 ^b^	Low
Mittal et al., 2006 [20]	India	Asian	Adult	PCR-RFLP	Healthy subjects	16	82	32	30	72	48	0.10	Low
Otzurk et al., 2008 [21]	Turkey	Caucasian	Children	PCR-RFLP	Healthy subjects & 1st-time stone formers	27	4	9	28	0	12	40 ^b^	Moderate
Tsai et al., 2002 [22]	Taiwan	Asian	Adult	PCR-RFLP	Healthy subjects	135	18	0	101	4	0	0.04	Low
**(*VDR* ApaI)**	**Country**	**Ethnicity**	**Age group**	**Genotyping method**	**Controls**	**Cases (n)**			**Controls (n)** ^**a**^				**Bias Risk (QUIPS)**
						**AA**	**Aa**	**aa**	**AA**	**Aa**	**aa**	**X**^**2**^ **HWE**	
Franco et al., 2007 [23]	Spain	Caucasian	Adult	PCR-RFLP	Healthy subjects	11	29	11	7	9	4	0.13	Low
Rendina et al., 2004 [24]	Italy	Caucasian	Adult	PCR-RFLP	Healthy subjects	43	87	29	37	68	19	1.80	Moderate
Wang et al., 2012 [7]	China	Asian	Adult	PCR-RFLP	Healthy subjects & 1st-time stone formers	2	36	67	46	195	209	0.003	Low
**(*VDR* BsmI)**	**Country**	**Ethnicity**	**Age group**	**Genotyping method**	**Controls**	**Cases (n)**			**Controls (n)** ^**a**^				**Bias Risk (QUIPS)**
						**BB**	**Bb**	**bb**	**BB**	**Bb**	**bb**	**X**^**2**^ **HWE**	
Franco et al., 2007 [23]	Spain	Caucasian	Adult	PCR-RFLP	Healthy subjects	5	25	21	5	9	6	0.19	Low
Mossetti et al., 2003 [25]	Italy	Caucasian	Adult	PCR-RFLP	Healthy subjects	54	94	72	38	46	30	4.07 ^b^	Low
Mossetti et al., 2004 [26]	Italy	Caucasian	Adult	PCR-RFLP	Healthy subjects	40	46	24	40	56	31	1.64	Low
Rendina et al., 2004 [24]	Italy	Caucasian	Adult	PCR-RFLP	Healthy subjects	47	69	43	39	56	29	1.02	Moderate
**(*VDR* FokI)**	**Country**	**Ethnicity**	**Age group**	**Genotyping method**	**Controls**	**Cases (n)**			**Controls (n)** ^**a**^				**Bias Risk (QUIPS)**
						**FF**	**Ff**	**ff**	**FF**	**Ff**	**ff**	**X**^**2**^ **HWE**	
Basiri et al., 2012 [27]	Iran	Asian	Adult	PCR	Healthy subjects	54	42	6	36	27	43	25.3 ^b^	Low
Liu et al., 2007 [28]	Taiwan	Asian	Adult	PCR-RFLP	Healthy subjects & 1st-time stone formers	40	60	25	58	116	57	0.04	Low
Mossetti et al., 2004 [26]	Italy	Caucasian	Adult	PCR-RFLP	Healthy subjects	43	47	20	53	55	19	0.57	Low
Rendina et al., 2004 [24]	Italy	Caucasian	Adult	PCR-RFLP	Healthy subjects	69	68	22	53	55	16	0.09	Moderate
**(*VDR* TaqI)**	**Country**	**Ethnicity**	**Age group**	**Genotyping method**	**Controls**	**Cases (n)**			**Controls (n)** ^**a**^				**Bias Risk (QUIPS)**
						**TT**	**Tt**	**tt**	**TT**	**Tt**	**tt**	**X**^**2**^ **HWE**	
Aykan et al., 2016 [18]	Turkey	Caucasian	Adult	PCR-RFLP	Healthy subjects & 1st-time stone formers	28	24	26	66	86	15	3.08	Low
Basiri et al., 2012 [27]	Iran	Asian	Adult	PCR	Healthy subjects	41	50	11	52	37	17	4.97 ^b^	Low
Franco et al., 2007 [23]	Spain	Caucasian	Adult	PCR-RFLP	Healthy subjects	21	26	4	6	9	5	0.19	Low
Jawad et al., 2020 [19]	Iraq	Asian	Adult	PCR-RFLP	Healthy subjects & 1st-time stone formers	48	33	19	55	60	12	0.57	Low
Mossetti et al., 2003 [25]	Italy	Caucasian	Adult	PCR-RFLP	Healthy subjects	80	104	36	35	66	13	4.68 ^b^	Low
Mossetti et al., 2004 [26]	Italy	Caucasian	Adult	PCR-RFLP	Healthy subjects	21	53	36	21	68	38	1.04	Low
Rendina et al., 2016 [29]	Italy	Caucasian	Adult	PCR-RFLP	Healthy subjects	186	158	28	31	44	13	0.17	Low
Seyhan et al., 2007 [30]	Turkey	Caucasian	Children	PCR-RFLP	Healthy subjects & 1st-time stone formers	9	15	16	13	25	2	4.96 ^b^	Low

^a^ Genotypes shown are for controls from healthy subjects

^b^ deviation from HWE (X^2^ >3.84).

HWE: Hardy–Weinberg equilibrium; PCR–RFLP: polymerase chain reaction–restriction fragment length polymorphism; QUIPS: quality in prognosis studies; VDR: vitamin D receptor.

**Table 3:** Risk of bias from individual studies included in the meta–analysis.

Study	Genetic polymorphisms	Risk of Bias (QUIPS)						Overall Bias Risk (QUIPS)
		Study participation	Study attrition	Prognostic factor measurement	Outcome measurement	Study confounding	Statistical analysis	
Aykan et al., 2016 [18]	Urokinase (ApaLI) & *VDR* (TaqI)	Moderate	Low	Low	Low	Moderate	Low	Low
Basiri et al., 2012 [27]	*VDR* (FokI, TaqI)	Low	Low	Low	Moderate	Moderate	Low	Low
Franco et al., 2007 [23]	*VDR* (ApaI, BsmI, TaqI)	Low	Low	Low	Moderate	Moderate	Low	Low
Jawad et al., 2020 [19]	Urokinase (ApaLI) & *VDR* (TaqI)	Moderate	Low	Low	Low	Moderate	Low	Low
Liu et al., 2007 [28]	*VDR* (FokI)	Low	Low	Low	Moderate	Moderate	Low	Low
Mittal et al., 2006 [20]	Urokinase (ApaLI)	Low	Low	Low	Low	Moderate	Low	Low
Mossetti et al., 2003 [25]	*VDR* (BsmI, TaqI)	Low	Low	Low	Moderate	Low	Low	Low
Mossetti et al., 2004 [26]	*VDR* (BsmI, FokI, TaqI)	Moderate	Low	Low	Moderate	Low	Low	Low
Otzurk et al., 2008 [21]	Urokinase (ApaLI)	Moderate	Low	Low	Moderate	Moderate	Low	Moderate
Rendina et al., 2004 [24]	*VDR* (ApaI, BsmI, FokI)	Moderate	Low	Low	Moderate	Moderate	Low	Moderate
Rendina et al., 2016 [29]	*VDR* (TaqI)	Low	Low	Low	Low	Moderate	Low	Low
Seyhan et al., 2007 [30]	*VDR* (TaqI)	Moderate	Low	Low	Low	Moderate	Low	Low
Tsai et al., 2002 [22]	Urokinase (ApaLI)	Low	Low	Low	Moderate	Moderate	Low	Low
Wang et al., 2012 [7]	*VDR* (ApaI)	Low	Low	Low	Moderate	Moderate	Low	Low

QUIPS: quality in prognosis studies; VDR: vitamin D receptor.

### Effect of urokinase gene polymorphism (ApaLI) on recurrent kidney stones

**Fig 2:**
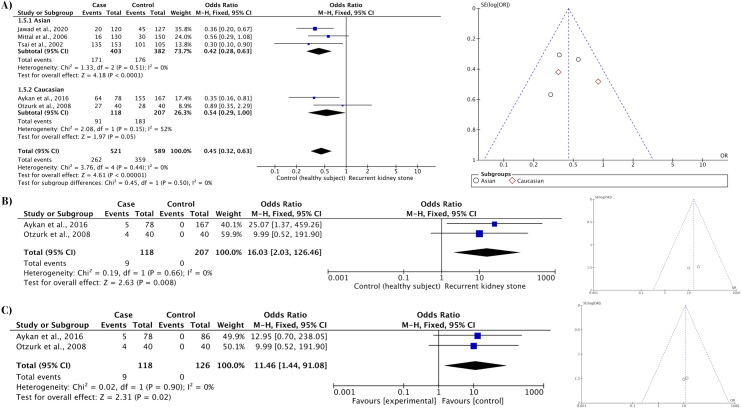
Forest and funnel plots for the ApaLI polymorphism. A) ApaLI polymorphism (recessive model) with healthy subject groups as controls. B) Sub–analysis of the ApaLI polymorphism (heterozygous model) among Caucasians with healthy subject groups as controls. C) Sub–analysis of the ApaLI polymorphism (heterozygous model) among Caucasians with first–time stone formers as controls.

The association between urokinase gene polymorphism (ApaLI*)* and recurrent kidney stones was analyzed using five models as shown in Table 4. Significant protective effects of the ApaLI polymorphism on recurrent kidney stones were observed among healthy subject groups in the recessive model (CC vs. CT/TT: OR 0.45, *P* < 0.01) (Table 4; Fig 2A); no significant associations were identified in the allelic frequencies, homozygous, heterozygous, and dominant models. Table 5 showed the association in the recessive model was still significant when restriction of larger studies was applied (CC vs. CT/TT: OR 0.40, *P* < 0.01), suggesting the lack of publication bias. A significant protective effect of this polymorphism was also apparent upon sub-analysis among Asian (CC vs. CT/TT: OR 0.42, *P* < 0.01) but not Caucasian healthy subject groups (CC vs. CT/TT: OR 0.54, *P* = 0.05) (Table 6; Fig 2A). However, sub-analysis among Caucasians revealed significantly higher risk of recurrent kidney stone development in the heterozygous model (CT vs. CC/TT: OR 16.03, *P* < 0.01) (Table 6; Fig 2B). No asymmetry was noted in the resultant funnel plot (Fig 2A and 2B), suggesting the lack of publication bias.

**Table 4:** Pooled OR analysis between the urokinase (ApaLI) and *VDR* (ApaI, BsmI, FokI, and TaqI) gene polymorphisms and recurrent kidney stones.

Gene Polymorphisms	N^a^	Allelic Frequencies Model		Homozygous Model		Heterozygous Model		Dominant Model		Recessive Model	
		OR	Model^b^	OR	Model^b^	OR	Model^b^	OR	Model^b^	OR	Model^b^
**Compared to healthy subjects**											
Urokinase (ApaLI)	5	0.55 (0.29–1.04)	Random	0.57 (0.26–1.24)	Random	2.16 (0.63–7.38)	Random	0.70 (0.24–2.05)	Random	**0.45 (0.32**–**0.63)**	Fixed
*VDR* (ApaI)	3	0.69 (0.46–1.03)	Random	0.44 (0.15–1.27)	Random	0.86 (0.64–1.17)	Fixed	**0.60 (0.42**–**0.85)**	Fixed	0.51 (0.21–1.26)	Random
*VDR* (BsmI)	4	0.87 (0.72–1.05)	Fixed	0.78 (0.55–1.11)	Fixed	1.00 (0.76–1.30)	Fixed	0.84 (0.62–1.14)	Fixed	0.85 (0.64–1.14)	Fixed
*VDR* (FokI)	4	1.36 (0.88–2.26)	Random	1.80 (0.66–4.90)	Random	1.08 (0.85–1.38)	Fixed	1.72 (0.65–4.56)	Random	1.27 (0.98–1.63)	Fixed
*VDR* (TaqI)	8	0.93 (0.70–1.25)	Random	0.82 (0.41–1.64)	Random	**0.72 (0.59**–**0.88)**	Fixed	0.71 (0.36–1.42)	Random	1.14 (0.93–1.41)	Fixed
**Compared to first time stone former**											
Urokinase (ApaLI)	3	0.59 (0.23–1.53)	Random	0.79 (0.25–2.46)	Random	3.01 (0.24–38.12)	Random	0.79 (0.50–1.26)	Fixed	0.61 (0.14–2.68)	Random
*VDR* (ApaI)	1	*Not Performed* ^*c*^									
*VDR* (BsmI)	0	*Not Performed* ^*c*^									
*VDR* (FokI)	0	*Not Performed* ^*c*^									
*VDR* (TaqI)	2	**0.69 (0.51–0.93)**	Fixed	**0.40 (0.22**–**0.74)**	Fixed	**0.62 (0.40**–**0.95)**	Fixed	**0.36 (0.21–0.64)**	Fixed	0.88 (0.58–1.33)	Fixed

^a^ number of studies.

^b^ if *P* value for the Q–test <0.10, the random–effects model was adopted; else the fixed–effects model was adopted.

^c^ analysis was not performed owing to the limited number of available studies (<2).

* significant results are marked in bold.

**Table 5:** Pooled OR analysis between the urokinase (ApaLI) and *VDR* (ApaI, BsmI, FokI, and TaqI) gene polymorphisms and recurrent kidney stones among larger studies (N > 200 samples).

Gene Polymorphisms	N^a^	Allelic Frequencies Model		Homozygous Model		Heterozygous Model		Dominant Model		Recessive Model	
		OR	Model^b^	OR	Model^b^	OR	Model^b^	OR	Model^b^	OR	Model^b^
**Compared to healthy subjects**											
Urokinase (ApaLI)	4	**0.47 (0.23–0.96)**	Random	0.45 (0.20–1.02)	Random	1.79 (0.49–6.56)	Random	0.57 (0.16–2.05)	Random	**0.40 (0.28–0.58)**	Fixed
*VDR* (ApaI)	2	0.67 (0.38–1.18)	Random	0.36 (0.06–2.09)	Random	0.81 (0.59–1.12)	Fixed	**0.58 (0.40–0.83)**	Fixed	0.44 (0.08–2.27)	Fixed
*VDR* (BsmI)	3	0.90 (0.74–1.09)	Fixed	0.82 (0.57–1.18)	Fixed	0.98 (0.75–1.30)	Fixed	0.86 (0.63–1.18)	Fixed	0.89 (0.66–1.20)	Fixed
*VDR* (FokI)	4	1.36 (0.88–2.26)	Random	1.80 (0.66–4.90)	Random	1.08 (0.85–1.38)	Fixed	1.72 (0.65–4.56)	Random	1.27 (0.98–1.63)	Fixed
*VDR* (TaqI)	6	0.96 (0.73–1.27)	Random	0.86 (0.44–1.67)	Random	**0.73 (0.60–0.90)**	Fixed	0.74 (0.38–1.45)	Random	1.16 (0.94–1.44)	Fixed
**Compared to first time stone former**											
Urokinase (ApaLI)	2	0.71 (0.20–2.56)	Random	1.02 (0.21–4.88)	Random	1.96 (0.08–46.92)	Random	0.86 (0.52–1.43)	Fixed	0.84 (0.10–6.87)	Random
*VDR* (ApaI)	1	*Not Performed* ^*c*^									
*VDR* (BsmI)	0	*Not Performed* ^*c*^									
*VDR* (FokI)	0	*Not Performed* ^*c*^									
*VDR* (TaqI)	3	**0.54 (0.31**–**0.94)**	Random	**0.27 (0.09**–**0.75)**	Random	**0.62 (0.42**–**0.91)**	Fixed	**0.29 (0.17**–**0.48)**	Fixed	0.76 (0.52–1.11)	Fixed

^a^ number of studies.

^b^ if *P* value for the Q–test <0.10, the random–effects model was adopted; else the fixed–effects model was adopted.

^c^ analysis was not performed owing to the limited number of available studies (<2).

* significant results are marked in bold.

**Table 6:** Sub–analysis between the urokinase (ApaLI) and VDR (ApaI, BsmI, FokI, and TaqI) gene polymorphisms and recurrent kidney stones among Asian and Caucasian ethnicity.

Gene Polymorphisms	Ethnicity	N^a^	Allelic Frequencies Model		Homozygous Model		Heterozygous Model		Dominant Model		Recessive Model	
			OR	Model^b^	OR	Model^b^	OR	Model^b^	OR	Model^b^	OR	Model^b^
**Compared to healthy subjects**												
Urokinase (ApaLI)	Asian	3	0.47 (0.18–1.24)	Random	0.42 (0.12–1.46)	Random	1.22 (0.33–4.57)	Random	0.55 (0.08–3.62)	Random	**0.42 (0.28**–**0.63)**	Fixed
	Caucasian	2	0.70 (0.28–1.74)	Random	0.82 (0.42–1.61)	Fixed	**16.03 (2.03**–**126.46)**	Fixed	0.91 (0.46–1.78)	Fixed	0.54 (0.29–1.0)	Fixed
*VDR* (ApaI)	Asian	1	*Not Performed* ^*c*^									
	Caucasian	2	0.86 (0.64–1.17)	Fixed	0.72 (0.38–1.38)	Fixed	1.08 (0.70–1.66)	Fixed	0.83 (0.47–1.46)	Fixed	0.80 (0.50–1.28)	Fixed
*VDR* (BsmI)	Asian	0	*Not Performed* ^*c*^									
	Caucasian	4	0.87 (0.72–1.05)	Fixed	0.78 (0.55–1.11)	Fixed	1.00 (0.76–1.30)	Fixed	0.84 (0.62–1.14)	Fixed	0.85 (0.64–1.14)	Fixed
*VDR* (FokI)	Asian	2	1.98 (0.80–4.88)	Random	3.97 (0.6–26.36)	Random	1.33 (0.61–2.94)	Random	3.66 (0.45–29.82)	Random	**1.70 (1.18–2.44)**	Fixed
	Caucasian	2	0.94 (0.73–1.21)	Fixed	0.86 (0.51–1.45)	Fixed	0.96 (0.67–1.35)	Fixed	0.85 (0.52–1.39)	Fixed	0.97 (0.68–1.37)	Fixed
*VDR* (TaqI)	Asian	2	0.91 (0.69–1.21)	Fixed	0.80 (0.45–1.44)	Fixed	0.98 (0.67–1.43)	Fixed	0.83 (0.24–2.89)	Random	0.93 (0.64–1.36)	Fixed
	Caucasian	6	0.94 (0.63–1.40)	Random	0.81 (0.31–2.09)	Random	**0.65 (0.51**–**0.82)**	Fixed	0.67 (0.28–1.63)	Random	1.25 (0.97–1.60)	Fixed
**Compared to first time stone former**												
Urokinase (ApaLI)	Asian	1	*Not Performed* ^*c*^									
	Caucasian	2	**0.37 (0.21**–**0.64)**	Fixed	**0.43 (0.19**–**0.99)**	Fixed	**11.46 (1.44**–**91.08)**	Fixed	0.48 (0.21–1.10)	Fixed	**0.29 (0.13**–**0.63)**	Fixed
*VDR* (ApaI)	Asian	1	*Not Performed* ^*c*^									
	Caucasian	0	*Not Performed* ^*c*^									
*VDR* (BsmI)	Asian	0	*Not Performed* ^*c*^									
	Caucasian	0	*Not Performed* ^*c*^									
*VDR* (FokI)	Asian	0	*Not Performed* ^*c*^									
	Caucasian	0	*Not Performed* ^*c*^									
*VDR* (TaqI)	Asian	1	*Not Performed* ^*c*^									
	Caucasian	2	**0.44 (0.30**–**0.63)**	Fixed	**0.19 (0.09**–**0.41)**	Fixed	0.59 (0.35–1.00)	Fixed	**0.19 (0.09**–**0.39)**	Fixed	**0.56 (0.33**–**0.94)**	Fixed

^a^ number of studies.

^b^ if *P* value for the Q–test <0.10, the random–effects model was adopted; else the fixed–effects model was adopted.

^c^ analysis was not performed owing to the limited number of available studies (<2).

* significant results are marked in bold.

Associations between the ApaLI gene polymorphism and recurrent kidney stones among first-time stone formers are presented in Table 4. Overall, no significant effect was detected in any of the five models. Restriction of larger studies also did not detect any significant effect among five models as shown in Table 5. However, sub-analysis among Caucasians revealed significant protective effects against recurrent kidney stones in allelic frequencies (C-allele vs. T-allele: OR 0.37, *P* < 0.01), homozygous (CC vs. TT: OR 0.43, *P* = 0.05), and recessive (CC vs. CT/TT: OR 0.29, *P* < 0.01) models as shown in Table 6. Conversely, a significantly higher risk of developing recurrent kidney stones was apparent in the heterozygous model (CT vs. CC/TT: OR 11.46, *P* = 0.02) among first-time Caucasian stone formers (Table 6; Fig 2C). The funnel plot did not indicate publication bias among eligible studies (Fig 2C).

### Effects of *VDR* gene polymorphisms on recurrent kidney stones

#### ApaI

**Fig 3:**
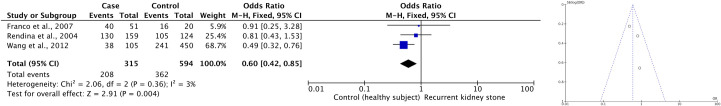
Forest plot and funnel plot of the *VDR* ApaI polymorphism (dominant model) with healthy subject groups as controls.

Table 4 shows the associations between the *VDR* ApaI polymorphism and recurrent kidney stones. A significant protective effect against recurrent kidney stones was noted among healthy subject groups in the dominant model (CC/CT vs. TT: OR 0.60, *P* < 0.01) (Table 4; Fig 3). The funnel plot showed no indication of publication bias among eligible studies (Fig 3). The protective effect among healthy subject groups in the dominant model was also present when restriction of larger studies was applied as shown in Table 5 (CC/CT vs. TT: OR 0.58, *P* < 0.01). However, sub-analysis among Caucasians revealed no significant association in any of the five models (Table 6). Sub-analyses among Asian ethnicity and first-time stone former groups were not performed owing to the limited number of available studies (Table 6).

#### BsmI

In this meta-analysis, associations between the *VDR* BsmI gene polymorphism and recurrent kidney stones were not statistically significant in the allelic frequencies, homozygous, heterozygous, dominant, and recessive models (Tables 4 and 5). Sub-analyses among Asian ethnicity and first-time stone former groups were also not performed owing to the limited number of available studies (Table 6).

#### FokI

**Fig 4:**
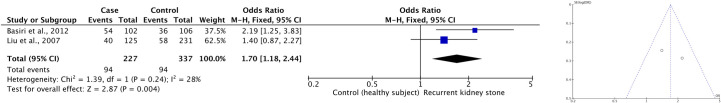
Forest and funnel plots of the *VDR* FokI polymorphism (recessive model) with healthy subject groups as controls.

Associations between the *VDR* FokI polymorphism and recurrent kidney stone were not statistically significant in any models with healthy subject groups (Tables 4 and 5). However, significant association was detected in the recessive model (FF vs. Ff/ff: OR 1.70, *P* < 0.01) among Asians as shown in Table 6 and Fig 4. The funnel plot did not exhibit any significant asymmetry (Fig 4). Comparison with first-time stone formers was not performed owing to the limited number of available studies.

#### TaqI

**Fig 5:**
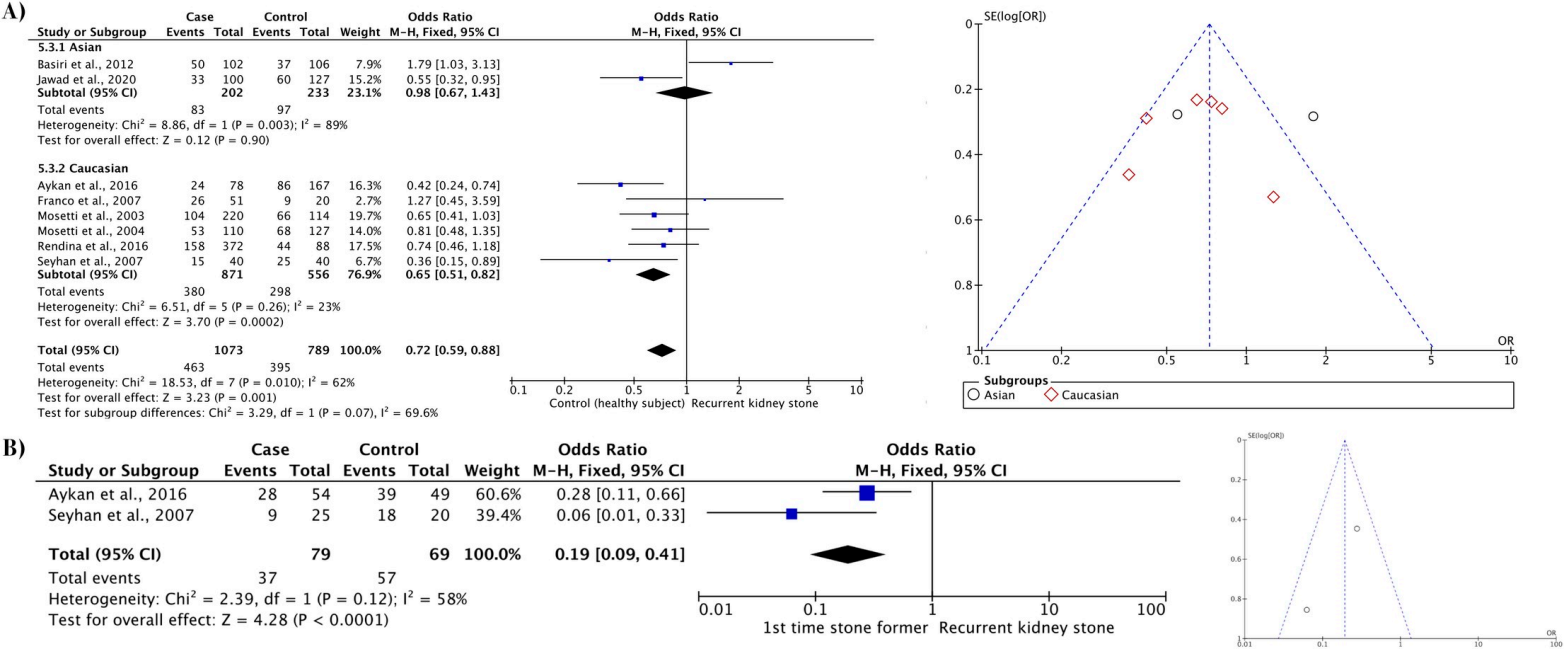
Forest and funnel plots of the *VDR* TaqI polymorphism. A) TaqI polymorphism (heterozygous model) with healthy subject groups as controls. B) Sub–analysis of the TaqI polymorphism (homozygous model) among Caucasians with first–time stone formers as controls.

The TaqI gene polymorphism exhibited significant protective association against recurrent kidney stones in the heterozygous model (Tt vs. TT/tt: OR 0.72, *P* < 0.01) as shown in Table 4. Table 5 showed the association in the heterozygous model was still significant when restriction of larger studies was applied (Tt vs. TT/tt: OR 0.73, *P* < 0.01), suggesting the lack of publication bias. This protective association was also observed upon sub-analysis of Caucasian (Tt vs. TT/tt: OR 0.65, *P* < 0.01) but not Asian populations (Tt vs. TT/tt: OR 0.98, *P* = 0.90) as shown in Table 6 and Fig 5A. The funnel plot did not reveal any significant asymmetry (Fig 5A).

Comparison with first-time stone formers is shown in Table 4. Significant protective associations of the TaqI gene polymorphism were noted in the allelic frequencies (T-allele vs. t-allele: OR 0.54, *P* = 0.03), homozygous (TT vs. tt: OR 0.27, *P* = 0.01), heterozygous (Tt vs. TT/tt: OR 0.62, *P* = 0.01), and dominant (TT/Tt vs. tt: OR 0.29, *P* < 0.01) models. All of the protective associations remained clinically significant when restriction of larger studies was applied as shown in Table 5. The strongest protective association was observed in the homozygous model; a similar finding was also observed upon sub-analysis of Caucasian populations (TT vs. tt: OR 0.19, *P* < 0.01) as shown in Table 6 and Fig 5B.

### Effects of other gene polymorphisms on recurrent kidney stones

Variation in the number of *AR* gene CAG repeat and *ER* gene TA repeat polymorphisms were associated with increased susceptibility to recurrent kidney stones among males as shown in individual studies [31]. Several polymorphisms of the *CALCR* and *CASR* genes were also associated with recurrent kidney stones [32, 33, 35, 36]. Conversely, other studies found no associations between other *CASR* gene polymorphisms and kidney stone recurrence [34, 37] *SLC13A2* (HNaDC-1) gene polymorphism (I550V) may be associated with hypocitraturia in recurrent renal stone formers [51]. Prevalence of the *SLC13A2* gene polymorphic variant (rs11567842) was also higher in hypocitraturic recurrent calcium-oxalate stone formers [29]. Other polymorphisms, such as in the *CDH1*, *VEGF*, *IL18*, *IL1RA*, *MnSOD*, *ORAI1*, and *TAP* genes, have also been reported to be associated with kidney stone recurrence [38–42, 44, 52]. Alternatively, other studies found that *ALPL*, *CYP17*, *IGF2*, *EGFR*, *IL1B*, *IL6*, *ITPKC*, *MTNR1A*, *OPN*, *ODC*, osteocalcin, *RGS14*, *SAT1*, *TNFA*, *TRPV5*, and matrix Gla protein gene polymorphisms were not associated with kidney stone recurrence [9, 34, 38–43, 46, 47, 49, 50]. Meta-analysis was not possible for these gene polymorphisms owing to an insufficient number of studies for each variant.

## Discussion

The primary outcome of interest in our study was to identify the effects of any gene polymorphisms on recurrent kidney stones. Toward this end, meta-analysis of all eligible studies was performed. Our findings suggested that protective effects against recurrent kidney stones were afforded by urokinase-ApaLI (recessive model), *VDR*-ApaI (dominant model), and *VDR*-TaqI (heterozygous model) gene polymorphisms. Sub-analyses among Caucasian and Asian populations were also performed. The urokinase-ApaLI (heterozygous model) gene polymorphism was associated with increased risk of recurrent kidney stones among the Caucasian population. In comparison, the *VDR*-FokI (recessive model) gene polymorphism was associated with increased risk of recurrent kidney stones among the Asian population. However, the *VDR*-BsmI gene polymorphism was not significantly associated with recurrent kidney stones.

Our results regarding the urokinase (ApaLI) gene polymorphism revealed protective effects in the recessive model (CC vs. CT/TT) among healthy subject groups. Specifically, individuals that possessed the “T” allele (CT/TT) exhibited 2.22-fold higher incidence of recurrent kidney stones than that of individuals with the CC genotype. Our result was consistent with several other studies [18, 19, 21, 22]. However, the association was not statistically significant when compared to first-time stone formers. We also identified an increased risk of recurrent kidney stones among Caucasian stone formers carrying the heterozygous (CT) genotype, which was also consistent with several studies [18, 21]. In contrast, no significant association was observed in individuals of Asian ethnicity. Differences in environmental exposure and ethnic background may have contributed to this variance.

Functionally, the ApaLI polymorphism is crucial for the regulation of urokinase gene expression [19]. The urokinase enzyme exhibits proteolytic activity and prevents organization of the organic matrix in the urinary tract, thereby inhibiting the precipitation of minerals [18]. Thus, lower urokinase activity in the urinary tract will raise uromucoid levels, which may be involved in the formation of kidney stones by triggering matrix mineralization [20].

ApaLI polymorphism is a single nucleotide polymorphism located at the 3^’^-untranslated region (UTR) of urokinase gene [18]. The genetic variants in 3^’^-UTR may affect the ribonucleic acid (RNA) expression which influence risk of recurrent kidney stone formation. N4-acetylcytidine (ac4C) is a modified nucleoside that also affects RNA expression [57]. Recent researches suggest that ac4C is playing key role in several diseases, including recurrent kidney stone. ac4C helps to correctly read codons during translation and improves translation efficiency and the stability of mRNA [57].

In turn, *VDR* gene polymorphisms (ApaI, BsmI, TaqI, and FokI) may also influence VDR protein activity and expression, which play significant roles in kidney stone formation [7]. Vitamin D is an important component in mineral metabolism, including stimulation of intestinal absorption, bone resorption, and renal reabsorption of phosphate and calcium [7]. *VDR* gene polymorphisms may affect vitamin D signaling pathways, thus affecting kidney stone formation. Aside from *VDR* gene polymorphisms, prenatal vitamin D deficiency may also influences the risk of kidney stone formation. Study by Wang et al. [58] found that maternal vitamin D status were positively associated with neonatal vitamin D status. However our study did not assess the contribution of prenatal vitamin D deficiency toward the risk of recurrent kidney stone formation.

Our study revealed the protective association of the *VDR* ApaI gene polymorphism against recurrent kidney stones in the dominant model (AA/Aa vs. aa). This result was consistent with the study by Wang et al. [7] which revealed that the prevalence of the “a” allele was significantly higher in recurrent kidney stone formers. In comparison, our study found higher risk of recurrent kidney stones among Asian populations carrying the FF genotype (vs. Ff/ff genotypes) of the Fok1 polymorphism, which was consistent with a study by Basiri et al. [27]. The FokI gene polymorphism affects the transcriptional activity and order of the VDR protein, which lead to the initiation of pathological conditions [59]. Therefore, it is logical to presume a potential contribution of the FokI gene polymorphism in kidney stone recurrence as well.

The BsmI and TaqI gene polymorphisms are located near the end of the *VDR* gene or in the 3′ untranscribed region, respectively [25]. These polymorphisms has been hypothesized as being responsible for differences in VDR translational efficiency or messenger RNA stability, resulting in changes in VDR protein expression [25]. However, these polymorphisms do not modify the structure of the VDR protein [59]. Our meta-analysis did not identify any significant association of the BsmI gene polymorphism with recurrent kidney stones in any of the five models. In contrast, we found protective association of the TaqI polymorphism in the heterozygous model. This result was consistent with several studies that observed lower incidence of the Tt genotype among recurrent kidney stone formers in both adults and children [18, 30]. The incidence of the homozygous genotype TT (vs. tt) among Caucasians in the present meta-analysis was lower in the case groups than that in non-recurrent kidney stone formers. This result was also consistent with several studies in both adults and children [18, 30]. Functionally, the BsmI and TaqI gene polymorphisms have been suggested to contribute to the changes of urinary biochemical parameters in recurrent kidney stone formers [24, 25]. Rendina et al. [24] found that the BB genotype of the BsmI polymorphism was more frequent among fasting idiopathic hypercalciuric recurrent stone formers. Mossetti et al. [25] also observed a higher incidence of the BB genotype of the BsmI gene polymorphism and the tt genotype of the TaqI gene polymorphism among hypocitraturic recurrent stone formers. However, our meta-analysis was not able to perform further sub-analysis based on hypercalciuria or hypocitraturia status owing to the limited available data.

To our knowledge, this meta-analysis represents the only study to assess the association between genetic polymorphisms and recurrent kidney stones. We performed quality control by performing meta-analysis with restriction of larger studies (N > 200 cases) as performed by Xu et al. [16]. All of the significant associations in this meta-analysis remained significant after restriction of larger studies was applied, suggesting lack of publication bias. We also performed sub-group analysis on possible sources of confounding factors as performed by Jiang et al. in their meta-analysis [15]. However, this meta-analysis had several imitations. The number of studies that could be added in the meta-analysis was limited. Sub-group analysis of ethnicity was not performed in several genetic polymorphisms (Table 6) due to limited number of studies. Some other sub-group analyses were also not possible owing to the limited number of available studies. Trans-trait meta-analysis which was performed for genetic meta-analysis such as by Wu et al. cannot be performed in this study [60]. Biochemical parameters affecting kidney stone formation could not be assessed owing to insufficient available data. Other risk factors associated with kidney stone formation including sex, lifestyle, and dietary habits were not controlled. The studies included in the meta-analysis were limited to those in English only. This may have excluded eligible studies in other languages. Further studies to construct a standard prediction model with receiver operating characteristic (ROC) curve analysis [61] for both urokinase and *VDR* gene polymorphisms are needed. A multi-modal deep convolutional neural network [62] is also a promising method to predict risk of recurrent kidney stone formation in the future.

## Conclusion

This meta-analysis revealed existing gaps in our understanding of how genetic polymorphisms affect recurrent kidney stones. Our meta-analysis showed that urokinase-ApaLI (recessive model), *VDR*-ApaI (dominant model), and *VDR*-TaqI (heterozygous model) polymorphisms were significantly associated with decreased risk of recurrent kidney stones. Conversely, the urokinase-ApaLI (heterozygous model) gene polymorphism was significantly associated with increased risk of recurrent kidney stones among Caucasian populations whereas the *VDR*-FokI (recessive model) gene polymorphism was associated with increased risk of recurrent kidney stones among Asian populations.

## Supporting information

S1 Checklist PRISMA 2009 checklist. (PDF)

S2 Checklist Meta-analysis on genetic association studies checklist. (DOCX)

## Abbreviations

HWE: Hardy–Weinberg equilibrium
OR: odds ratio
PCR-RFLP: polymerase chain reaction-restriction fragment length polymorphism
QUIPS: Quality in Prognostic Studies
VDR: vitamin D receptor

## References

[1] Trinchieri A. Epidemiology of urolithiasis: an update. Clin Cases Miner Bone Metab. 2008; 5: 101–106. 22460989 PMC2781200

[2] Scales CD Jr, Smith AC, Hanley JM, Saigal CS, Urologic Diseases in America Project. Prevalence of kidney stones in the United States. Eur Urol. 2012; 62: 160–165. 10.1016/j.eururo.2012.03.052 22498635 PMC3362665

[3] Uribarri J, Oh MS, Carroll HJ. The first kidney stone. Ann Intern Med. 1989; 111: 1006–1009. 10.7326/0003-4819-111-12-1006 2688503

[4] Liu Y, Chen Y, Liao B, et al. Epidemiology of urolithiasis in Asia. Asian J Urol. 2018; 5: 205–214. 10.1016/j.ajur.2018.08.007 30364478 PMC6197415

[5] Dion M, Ankawi G, Chew B, et al. CUA guideline on the evaluation and medical management of the kidney stone patient—2016 update. Can Urol Assoc J. 2016; 10: E347–E358. 10.5489/cuaj.4218 28096919 PMC5234401

[6] Curhan GC, Willett WC, Rimm EB, Stampfer MJ. A prospective study of dietary calcium and other nutrients and the risk of symptomatic kidney stones. N Engl J Med. 1993; 328: 833–838. 10.1056/NEJM199303253281203 8441427

[7] Pritchard JK, Cox NJ. The allelic architecture of human disease genes: common disease-common variant… or not? Hum Mol Genet. 2002; 11: 2417–2423. 10.1093/hmg/11.20.2417 12351577

[8] Wang S, Wang X, Wu J, et al. Association of vitamin D receptor gene polymorphism and calcium urolithiasis in the Chinese Han population. Urol Res. 2012; 40: 277–284. 10.1007/s00240-011-0438-y 22116536

[9] Khaleel A, Wu MS, Wong HSC, et al. A single nucleotide polymorphism (rs4236480) in TRPV5 calcium channel gene is associated with stone multiplicity in calcium nephrolithiasis patients. Mediators Inflamm. 2015; 2015: 375427. 10.1155/2015/375427 26089600 PMC4452106

[10] Mittal RD, Bid HK, Manchanda PK, Kapoor R. Predisposition of genetic polymorphism with the risk of urolithiasis. Indian J Clin Biochem. 2008; 23: 106–116. 10.1007/s12291-008-0027-1 23105735 PMC3453093

[11] Liberati A, Altman DG, Tetzlaff J, et al. The PRISMA statement for reporting systematic reviews and meta-analyses of studies that evaluate healthcare interventions: explanation and elaboration. BMJ. 2009; 339: b2700. 10.1136/bmj.b2700 19622552 PMC2714672

[12] Hayden JA, van der Windt DA, Cartwright JL, Côté P, Bombardier C. Assessing bias in studies of prognostic factors. Ann Intern Med. 2013; 158: 280–286. 10.7326/0003-4819-158-4-201302190-00009 23420236

[13] Grooten WJA, Tseli E, Äng BO, et al. Elaborating on the assessment of the risk of bias in prognostic studies in pain rehabilitation using QUIPS-aspects of interrater agreement. Diagnostic Progn Res. 2019; 3: 5. 10.1186/s41512-019-0050-0 31093575 PMC6460536

[14] Moons KGM, de Groot JAH, Bouwmeester W, et al. Critical appraisal and data extraction for systematic reviews of prediction modelling studies: the CHARMS checklist. PLoS Med. 2014; 11: e1001744. 10.1371/journal.pmed.1001744 25314315 PMC4196729

[15] Jiang L, Wang K, Lo K, et al. Sex-Specific Association of Circulating Ferritin Level and Risk of Type 2 Diabetes: A Dose-Response Meta-Analysis of Prospective Studies. J Clin Endocrinol Metab. 2019 10 1;104(10):4539–4551. 10.1210/jc.2019-00495 31074789

[16] Xu MQ, Ye Z, Hu FB, He L. Quantitative assessment of the effect of angiotensinogen gene polymorphisms on the risk of coronary heart disease. Circulation. 2007 9 18;116(12):1356–66. 10.1161/CIRCULATIONAHA.107.728857 17846284

[17] Rodriguez S, Gaunt TR, Day INM. Hardy–Weinberg equilibrium testing of biological ascertainment for Mendelian randomization studies. Am J Epidemiol. 2009; 169: 505–514. 10.1093/aje/kwn359 19126586 PMC2640163

[18] Aykan S, Tuken M, Gunes S, et al. ApaL1 urokinase and Taq1 vitamin D receptor gene polymorphisms in first-stone formers, recurrent stone formers, and controls in a Caucasian population. Urolithiasis. 2016; 44: 109–115. 10.1007/s00240-015-0813-1 26275878

[19] Jawad ZN, Awad W. Association of urokinase and Vitamin D receptor genes SNPs and urolithiasis in an Iraqi population. Meta Gene. 2020; 24: 100679.

[20] Mittal RD, Bid HK, Kumar A, Bhandari M. Association of urokinase gene 3′-UTR polymorphism with calcium oxalate nephrolithiasis. J Endourol. 2006; 20: 157–160. 10.1089/end.2006.20.157 16509805

[21] Ozturk M, Kordan Y, Cangul H, et al. Association of urokinase gene 3′-UTR T/C polymorphism with calcium oxalate urolithiasis in children. Int Urol Nephrol. 2008; 40: 563–568. 10.1007/s11255-008-9335-x 18240004

[22] Tsai FJ, Lin CC, Lu HF, Chen HY, Chen WC. Urokinase gene 3′-UTR T/C polymorphism is associated with urolithiasis. Urology. 2002; 59: 458–461. 10.1016/s0090-4295(01)01576-x 11880102

[23] Moyano Franco MJ, de Tejada Romero MJG, García Lozano R, et al. Changes in bone mineral metabolism in patients with calcium kidney stone disease and polymorphism of vitamin D receptor. Preliminary results. Nefrologia. 2007; 27: 694–703. http://www.embase.com/search/results?subaction=viewrecord&from=export&id=L351604848. 18336098

[24] Rendina D, Mossetti G, Viceconti R, et al. Association between vitamin D receptor gene polymorphisms and fasting idiopathic hypercalciuria in recurrent stone-forming patients. Urology. 2004; 64: 833–838. 10.1016/j.urology.2004.05.013 15491743

[25] Mossetti G, Vuotto P, Rendina D, et al. Association between vitamin D receptor gene polymorphisms and tubular citrate handling in calcium nephrolithiasis. J Intern Med. 2003; 253: 194–200. 10.1046/j.1365-2796.2003.01086.x 12542560

[26] Mossetti G, Rendina D, Viceconti R, et al. The relationship of 3′ vitamin D receptor haplotypes to urinary supersaturation of calcium oxalate salts and to age at onset and familial prevalence of nephrolithiasis. Nephrol Dial Transplant. 2004; 19: 2259–2265. 10.1093/ndt/gfh273 15213319

[27] Basiri A, Shakhssalim N, Houshmand M, et al. Coding region analysis of vitamin D receptor gene and its association with active calcium stone disease. Urol Res. 2012; 40: 35–40. 10.1007/s00240-011-0399-1 21814771

[28] Liu CC, Huang CH, Wu WJ, et al. Association of vitamin D receptor (Fok-I) polymorphism with the clinical presentation of calcium urolithiasis. BJU Int. 2007; 99: 1534–1538. 10.1111/j.1464-410X.2007.06792.x 17419705

[29] Rendina D, De Filippo G, Gianfrancesco F, et al. Evidence for epistatic interaction between *VDR* and *SLC13A2* genes in the pathogenesis of hypocitraturia in recurrent calcium oxalate stone formers. J Nephrol. 2017; 30: 411–418. 10.1007/s40620-016-0348-8 27639591

[30] Seyhan S, Yavascaoglu I, Kilicarslan H, Dogan HS, Kordan Y. Association of vitamin D receptor gene TaqI polymorphism with recurrent urolithiasis in children. Int J Urol. 2007; 14: 1060–1062. 10.1111/j.1442-2042.2007.01899.x 18036039

[31] Chen WC, Wu HC, Lin WC, et al. The association of androgen- and oestrogen-receptor gene polymorphisms with urolithiasis in men. BJU Int. 2001; 88: 432–436. 10.1046/j.1464-410x.2001.02319.x 11564035

[32] Chen WC, Wu HC, Lu HF, Chen HY, Tsai FJ. Calcitonin receptor gene polymorphism: A possible genetic marker for patients with calcium oxalate stones. Eur Urol. 2001; 39: 716–719. 10.1159/000052532 11464063

[33] Shakhssalim N, Basiri A, Houshmand M, et al. Genetic polymorphisms in calcitonin receptor gene and risk for recurrent kidney calcium stone disease. Urol Int. 2014; 92: 356–362. 10.1159/000353348 24296906

[34] Chen WC, Chou WH, Chu HW, et al. The rs1256328 (ALPL) and rs12654812 (RGS14) polymorphisms are associated with susceptibility to calcium nephrolithiasis in a Taiwanese population. Sci Rep. 2019; 9: 17296. 10.1038/s41598-019-53261-8 31754202 PMC6872875

[35] Shakhssalim N, Kazemi B, Basiri A, et al. Association between calcium-sensing receptor gene polymorphisms and recurrent calcium kidney stone disease: A comprehensive gene analysis. Scand J Urol Nephrol. 2010; 44: 406–412. 10.3109/00365599.2010.497770 20602573

[36] Vezzoli G, Terranegra A, Aloia A, et al. Decreased transcriptional activity of calcium-sensing receptor gene promoter 1 is associated with calcium nephrolithiasis. J Clin Endocrinol Metab. 2013; 98: 3839–3847. 10.1210/jc.2013-1834 23864702 PMC3763974

[37] Chou YH, Woon PY, Chen WC, et al. A genetic polymorphism (rs17251221) in the calcium-sensing receptor gene (*CASR*) is associated with stone multiplicity in calcium nephrolithiasis. PLoS One. 2011; 6: e25227. 10.1371/journal.pone.0025227 21966463 PMC3178627

[38] Tsai FJ, Wu HC, Chen HY, et al. Association of E-cadherin gene 3′-UTR C/T polymorphism with calcium oxalate stone disease. Urol Int. 2003; 70: 278–281. 10.1159/000070135 12740491

[39] Chen WC, Chen HY, Wu HC, et al. Vascular endothelial growth factor gene polymorphism is associated with calcium oxalate stone disease. Urol Res. 2003; 31: 218–222. 10.1007/s00240-003-0325-2 12719950

[40] Lai KC, Lin WY, Man KM, et al. Association of interleukin-18 gene polymorphisms with calcium oxalate kidney stone disease. Scand J Urol Nephrol. 2010; 44: 20–26. 10.3109/00365590903449332 20017708

[41] Chen WC, Wu HC, Chen HY, et al. Interleukin-1β gene and receptor antagonist gene polymorphisms in patients with calcium oxalate stones. Urol Res. 2001; 29: 321–324. 10.1007/s002400100193 11762793

[42] Çoker Gurkan A, Arisan S, Arisan ED, Sönmez NC, Palavan Ünsal N. Association between IL-1RN VNTR, IL-1β −511 and IL-6 (−174, −572, −597) gene polymorphisms and urolithiasis. Urol Int. 2013; 91: 220–226. 10.1159/000345786 23363559

[43] Kan WC, Chou YH, Chiu SJ, et al. Study of the association between ITPKC genetic polymorphisms and calcium nephrolithiasis. Biomed Res Int. 2014; 2014: 397826. 10.1155/2014/397826 24800221 PMC3988947

[44] Tugcu V, Ozbek E, Aras B, et al. Manganese superoxide dismutase (Mn-SOD) gene polymorphisms in urolithiasis. Urol Res. 2007; 35: 219–224. 10.1007/s00240-007-0103-7 17628794

[45] Esposito T, Rendina D, Aloia A, et al. The melatonin receptor 1A (MTNR1A) gene is associated with recurrent and idiopathic calcium nephrolithiasis. Nephrol Dial Transplant. 2012; 27: 210–218. 10.1093/ndt/gfr216 21652546

[46] Tugcu V, Simsek A, Tarhan T, et al. *OPN* gene polymorphism (Ala250) and lower serum OPN levels are associated with urolithiasis. Ren Fail. 2013; 35: 825–829. 10.3109/0886022X.2013.794431 23692545

[47] Yamate T, Tsuji H, Amasaki N, et al. Analysis of osteopontin DNA in patients with urolithiasis. Urol Res. 2000; 28: 159–166. 10.1007/s002400000112 10929424

[48] Chou YH, Juo SHH, Chiu YC, et al. A polymorphism of the *ORAI1* gene is associated with the risk and recurrence of calcium nephrolithiasis. J Urol. 2011; 185: 1742–1746. 10.1016/j.juro.2010.12.094 21420116

[49] Ҫoker-Gürkan A, Arisan S, Arisan ED, Ünsal NP. Lack of evidence for the association of ornithine decarboxylase (+316 G>A), spermidine/spermine acetyl transferase (−1415 T>C) gene polymorphisms with calcium oxalate stone disease. Biomed Rep. 2014; 2: 69–74. 10.3892/br.2013.184 24649071 PMC3917696

[50] Chen WC, Chen HY, Wu JY, Chen YT, Tsai FJ. Osteocalcin gene HindIII polymorphism is not correlated with calcium oxalate stone disease. Urol Res. 2001; 29: 98–101. 10.1007/s002400100169 11396736

[51] Okamoto N, Aruga S, Matsuzaki S, et al. Associations between renal sodium-citrate cotransporter (hNaDC-1) gene polymorphism and urinary citrate excretion in recurrent renal calcium stone formers and normal controls. Int J Urol. 2007; 14: 344–349. 10.1111/j.1442-2042.2007.01554.x 17470169

[52] Huang SH, Chen RH, Wan L, Tsai FJ, Chen WC. *TAP2* gene Msp-I polymorphism might be associated with calcium oxalate stone disease. Urol Int. 2005; 75: 264–268. 10.1159/000087806 16215317

[53] Tsai FJ, Lu HF, Yeh LS, Hsu CD, Chen WC. Lack of evidence for the association of tumor necrosis factor-alpha gene promoter polymorphism with calcium oxalate stone and bladder cancer patients. Urol Res. 2001; 29: 412–416. 10.1007/s002400100219 11828995

[54] Li X, Dang X, Cheng Y, et al. Common variants in *ALPL* gene contribute to the risk of kidney stones in the Han Chinese population. Genet Test Mol Biomarkers. 2018; 22: 187–192. 10.1089/gtmb.2017.0208 29489416

[55] Gao B, Yasui T, Itoh Y, et al. A polymorphism of matrix Gla protein gene is associated with kidney stones. J Urol. 2007; 177: 2361–2365. 10.1016/j.juro.2007.01.118 17509359

[56] Lu X, Gao B, Liu Z, et al. A polymorphism of matrix Gla protein gene is associated with kidney stone in the Chinese Han population. Gene. 2012;511(2):127–130. 10.1016/j.gene.2012.09.112 23046575

[57] Jin G, Xu M, Zou M, Duan S. The Processing, Gene Regulation, Biological Functions, and Clinical Relevance of N4-Acetylcytidine on RNA: A Systematic Review. Mol Ther Nucleic Acids. 2020 6 5;20:13–24. 10.1016/j.omtn.2020.01.037 Epub 2020 Feb 8. ; PMCID: PMC7068197. 32171170 PMC7068197

[58] Wang X, Jiao X, Tian Y, Zhang J, Zhang Y, Li J, et al.; Shanghai Birth Cohort Study. Associations between maternal vitamin D status during three trimesters and cord blood 25(OH)D concentrations in newborns: a prospective Shanghai birth cohort study. Eur J Nutr. 2021 3 4. 10.1007/s00394-021-02528-w Epub ahead of print. . 33661376

[59] Uitterlinden AG, Fang Y, Van Meurs JBJ, Pols HAP, Van Leeuwen JPTM. Genetics and biology of vitamin D receptor polymorphisms. Gene. 2004; 338: 143–156. 10.1016/j.gene.2004.05.014 15315818

[60] Wu Y, Cao H, Baranova A, et al. Multi-trait analysis for genome-wide association study of five psychiatric disorders. Transl Psychiatry. 2020 6 30;10(1):209. 10.1038/s41398-020-00902-6 Erratum in: Transl Psychiatry. 2020 Jul 14;10(1):234. 32606422 PMC7326916

[61] Yu H, Pan R, Qi Y, Zheng Z, Li J, Li H, et al. LEPR hypomethylation is significantly associated with gastric cancer in males. Exp Mol Pathol. 2020 10;116:104493. 10.1016/j.yexmp.2020.104493 Epub 2020 Jul 11. . 32659237

[62] Liu M, Li F, Yan H, Wang K, Ma Y; Alzheimer’s Disease Neuroimaging Initiative, Shen L, et al. A multi-model deep convolutional neural network for automatic hippocampus segmentation and classification in Alzheimer’s disease. Neuroimage. 2020 3;208:116459. 10.1016/j.neuroimage.2019.116459 Epub 2019 Dec 16. . 31837471

